# Disitamab vedotin combined with pyrotinib as salvage treatment in Her-2-amplified treatment refractory metastatic colorectal cancer: a case report

**DOI:** 10.3389/fphar.2025.1431422

**Published:** 2025-02-20

**Authors:** Jianxin Chen, Jian Wang, Qinhong Zheng

**Affiliations:** ^1^ Department of Medical Oncology, Quzhou People′s Hospital, The Quzhou Affiliated Hospital of Wenzhou Medical University, Quzhou, Zhejiang, China; ^2^ The Third Clinical Medical College of Zhejiang Chinese Medical University, Hangzhou, Zhejiang, China

**Keywords:** Disitamab vedotin, pyrotinib, refractory metastatic colorectal cancer, HER-2 amplification, case report

## Abstract

**Background:**

Human epidermal growth factor receptor 2 (HER-2) amplification has been identified in approximately 3% of patients with metastatic colorectal cancer (mCRC). Owing to the lack of established anti-ERBB2 therapeutic approaches, mCRC patients with Her-2 amplification rarely receive targeted treatments. Moreover, conventional chemotherapy regimens are not ideal for these patients, leaving options in the advanced stage limited to best supportive care or participation in clinical drug trials.

**Case presentation:**

This report presents a case of a patient with Her-2-amplified refractory mCRC treated with a salvage regimen combining Disitamab Vedotin and Pyrotinib, resulting in a partial response and progression-free survival for 6 months, which is still ongoing.

**Conclusion:**

This case study suggests that the anti-Her-2 regimen involving Disitamab Vedotin and Pyrotinib may offer a potential salvage treatment option for patients with Her-2-amplified mCRC patients. However, further validation in larger cohorts is necessary in future studies.

## Introduction

Colorectal cancer (CRC) is the second most lethal malignancy; it accounts for 9.4% of all cancer-related deaths, with 20% of new cases presenting with metastatic disease at diagnosis (Li et al., 2021; Biller and Schrag, 2021; Strickler et al., 2022). Currently, surgery offers long-term benefits for a small subset of metastatic colorectal cancers (mCRC) with liver or lung metastases; however, treatment of the majority of patients is generally challenging, with only a 14% 5-year survival rate (Shin et al., 2023). Resultantly, current research and treatment approaches are increasingly focused on the identification and utilization of molecular subtypes. The intrinsic heterogeneity in molecular tumor subtypes, including variations in the expression of KRAS, NRAS, and BRAF genes, highlights the need for formulating varying treatment strategies for patients who remain unresponsive to systemic chemotherapy (Modest et al., 2019). Evidence from the available clinical trials and case reports suggests that personalized treatments based on the unique molecular characteristics of patients with mCRC could significantly enhance survival outcomes (Watanabe et al., 2023; Fakih et al., 2023; Krekeler et al., 2023). Therefore, for achieving better survival outcomes, molecular tumor profiling, the exploration of novel tumor biomarkers, and the development of new anti-tumor drugs are crucial.

Human Epidermal Growth Factor Receptor 2 (ERBB2), formerly known as HER-2, belongs to the family of epidermal growth factor receptors and plays an instrumental role in the regulation of cell proliferation and differentiation. ERBB2 amplification has been implicated in the carcinogenesis of diverse malignancies, including breast, lung, and gastric cancers, and in approximately 3% of cases of mCRC (Ross et al., 2018). This gene mutation can result in the overactivation of signaling pathways, including the MAPK/ERK and PI3K/Akt pathways, thereby promoting migration, proliferation, and adhesion of tumor cells (Budi et al., 2022). Recently, the U.S. Food and Drug Administration (FDA) approved the use of ERBB2-targeted drugs to treat breast and gastric cancers with ERBB2 amplification. However, no approved targeted therapy is available for ERBB2-amplified mCRC, particularly refractory mCRC (Strickler et al., 2022); hence, research into treatment options is actively underway. An objective response, a complete response or partial response, was observed in 20% to 30% of patients with ERBB2-amplified mCRC who received trastuzumab in combination with pertuzumab or lapatinib in the limited cohort phase II studies, HERACLES-A and Mypathway (Sartore-Bianchi et al., 2016; Meric-Bernstam et al., 2019). The drugs are both classic drugs targeting ERBB2 in clinical practice. This finding demonstrates the potential of anti-ERBB2 therapy for ERBB2-amplified mCRC.

**FIGURE 1:**
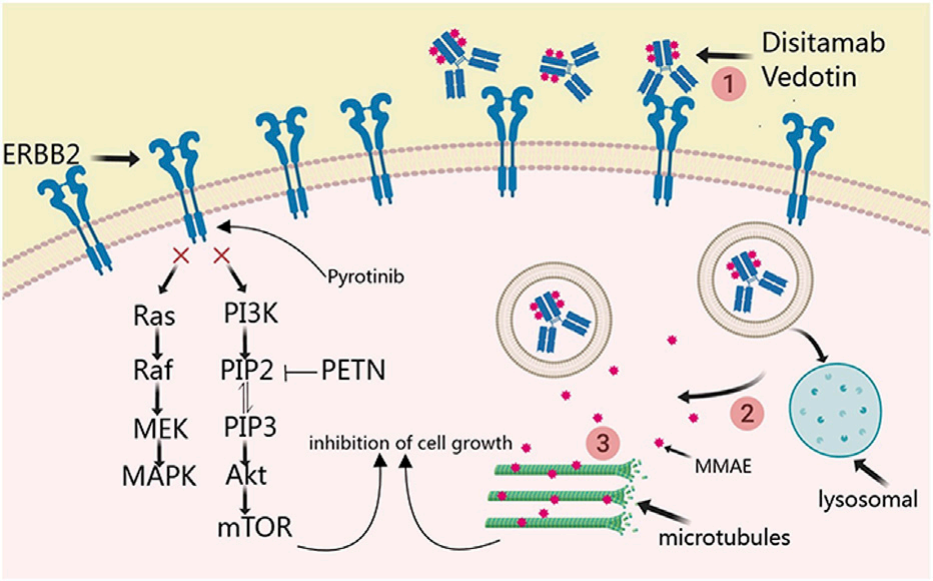
Principal mechanism of disitamab vedotin and pyrotinib in ERBB2-amplified mCRC.

Disitamab Vedotin is the first antibody-drug conjugate independently developed in China to target ERBB2 and has been approved by both the U.S. FDA and the China National Medical Products Administration (Deeks, 2021). In June 2021, Disitamab Vedotin was accorded approval for use in China for the treatment of patients with locally advanced or metastatic gastric cancer with ERBB2 overexpression, defined as IHC2+ or 3+. Subsequently, this drug was recommended for the treatment of other solid tumors exhibiting ERBB2 expression, including urothelial cancer, biliary tract cancer, non-small cell lung cancer, and breast cancer. The principal mechanism entails targeted delivery of the anticancer agent monomethyl auristatin E (MMAE) to HER2 receptor-expressing cells, thereby facilitating its anchorage to the cell membrane. This step is followed by MMAE endocytosis and release by active lysosomal enzymes. The released MMAE leads to the disruption of microtubules or tubulin structures within the cells, thereby resulting in tumor destruction (Shi et al., 2022). Pyrotinib, an irreversible dual pan-ErbB receptor tyrosine kinase inhibitor, inhibits the autophosphorylation of HER homodimers and heterodimers, thus effectively altering the Ras/Raf/MEK/MAPK and PI3K/Akt signaling cascades (Blair, 2018; Qi et al., 2023) (Figure 1). It targets EGFR and ERBB2 receptors. The current report presents a case of ERBB2 amplification in patients with mCRC that progressed despite treatment with trastuzumab and lapatinib. Following comprehensive discussions among the oncologists, an innovative anti-ERBB2 approach was implemented that combined Disitamab Vedotin with Pyrotinib, resulting in a partial response. This therapeutic strategy efficaciously arrested disease advancement and introduced a novel therapeutic option for ERBB2-amplified mCRC.

## Case presentation

**FIGURE 2:**
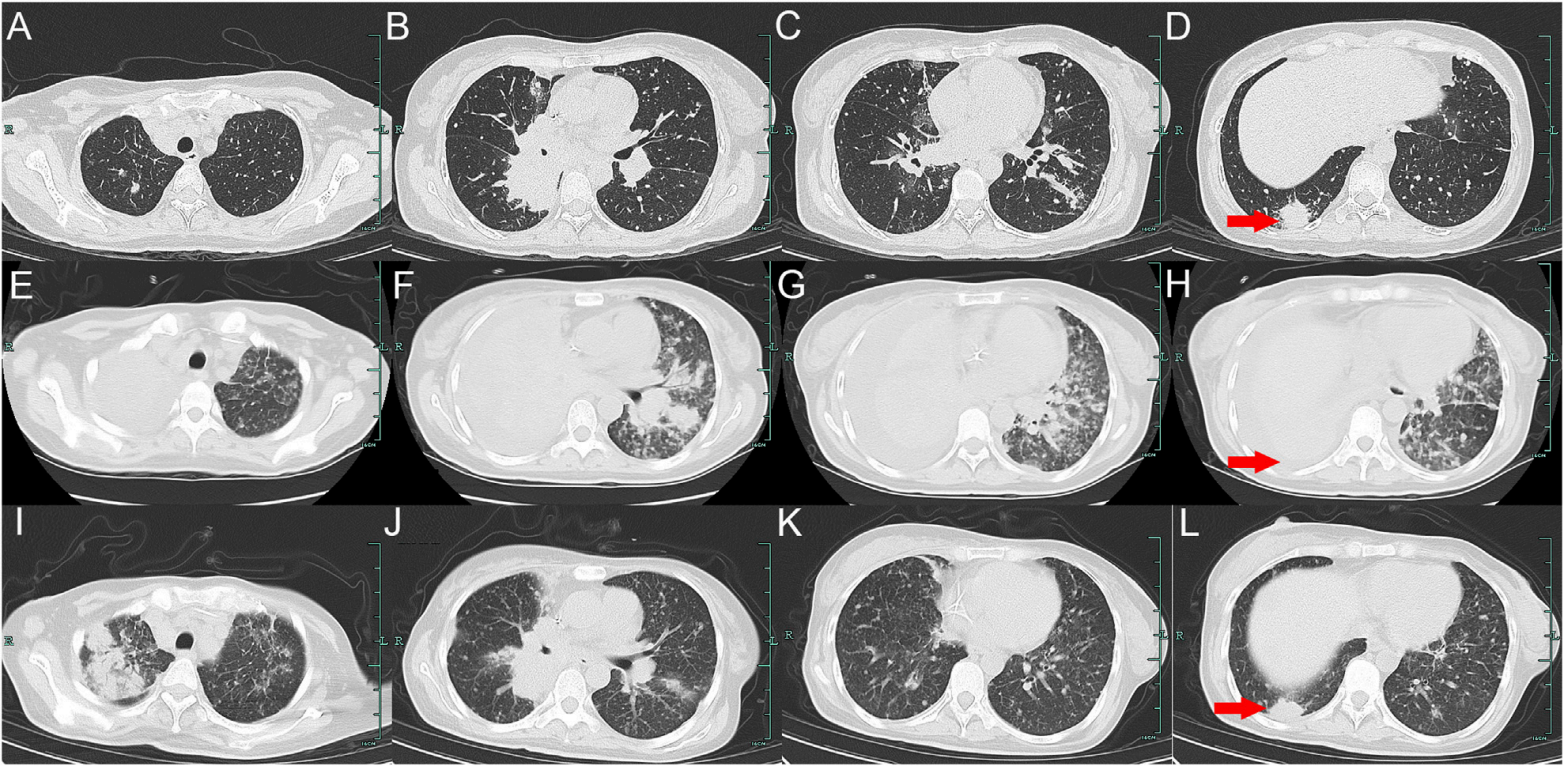
Variations in the primary lesions in the lung by chest CT scans during the treatment (red arrowheads). **(A–D)** were observed on 29 September 2023; **(E–H)** were observed on 1 November 2023; **(I–L)** were observed on 28 November 2023.

**FIGURE 3:**
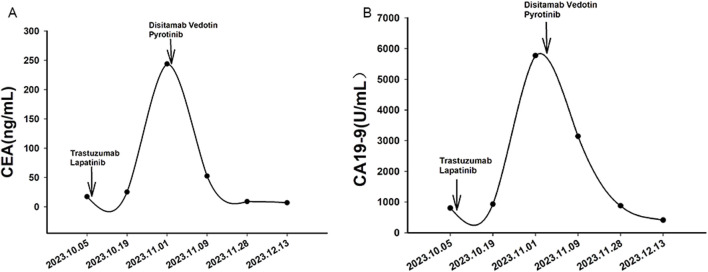
Variations in tumor markers CEA **(A)** and CA19-9 **(B)** during treatment. Arrows indicate the initiation of different treatment regimens. Trastuzumab and lapatinib were administered as salvage therapy starting from October 6, 2023, while Disitamab Vedotin and Pyrotinib were introduced on November 3, 2023.

**FIGURE 4:**
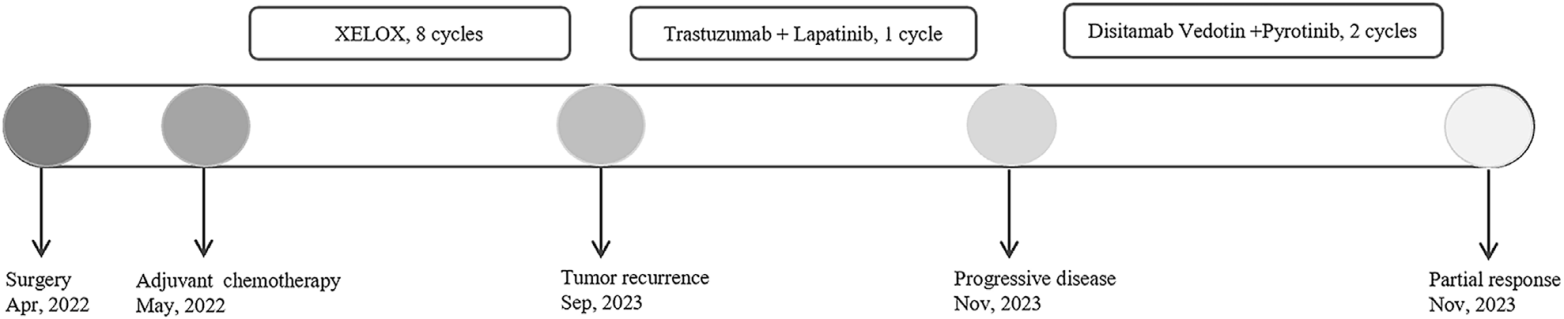
Summary of the treatment regimens and clinical features.

A 32-year-old Chinese woman with hemoptysis and fatigue persisting for over 2 months was admitted to our institution on 29 September 2023. She denied any history of smoking, alcohol consumption, or any related medical or family and received a left hemicolectomy for colon cancer at the Second Affiliated Hospital of Zhejiang University on 25 April 2022. Her postoperative pathology result revealed a moderately to poorly differentiated adenocarcinoma in the left colon, with invasion into the serosal layer. Lymph node metastases were found in 6 out of 20 examined lymph nodes. Immunohistochemistry findings are as follows: pMMR (positive); CDX-2 (positive); Her-2 (positive, 3+); Synaptophysin (partially weakly positive); CD56 (negative); and Ki-67 (80%). The results of the next-generation sequencing (NGS) are as follows: ERBB2 amplification (CNG: 16.6), RAS/RAF/PI3KCA wild-type; and microsatellite stable (MSS). Considering the results, a diagnosis of ERBB2-amplified mCRC was undertaken and pT3N2M0 staged, according to guidelines by the American Joint Committee on Cancer (AJCC) version 8th (Amin et al., 2017). Subsequently, the patient received adjuvant chemotherapy with XELOX regimens (oxaliplatin 130 mg/m^2^ on day 1, followed by oral capecitabine 1,000 mg/m^2^ twice a day from day 1 to day 14, every 3 weeks) for eight cycles. As the patient demonstrated symptoms of hemoptysis, a chest CT on 29 September 2023 was performed; it revealed multiple masses and nodular shadows in both lungs, and enlarged lymph nodes in various locations including the mediastinum, bilateral pulmonary hila, lower neck, and the left axillary region (Figures 2A–D). Furthermore, multiple lesions were detected in the right liver lobe, bilateral adrenal glands, lower abdominal region, lungs, and the brain of the patient. The patient’s Performance Status Score (PS) was assessed as 3. Based on the patient’s presentation, past medical history, and imaging examinations such as chest CT, a diagnosis of recurrent colorectal cancer (TNM stage IV) with multiple metastases was confirmed. For extending supportive care, a series of measures, including hemostatic and anti-infection treatments, phlegm reduction, acid suppression, analgesia, and nutritional support were administered. Based on the efficacy of the anti-ERBB2 regimen in the HERACLES-A study (Sartore-Bianchi et al., 2016), a combination of trastuzumab with lapatinib was administered as salvage treatment on 6 October 2023 (Trastuzumab 8 mg/kg on day 1, followed by 6 mg/kg, every 3 weeks, and oral lapatinib 1,000 mg per day). However, the patient was readmitted 3 weeks later on 1 November 2023, due to chest tightness, extreme fatigue, respiratory distress, and an oxygen saturation level of 70%. Repeated chest CT revealed multiple metastatic lesions in both lungs, indicating a progression from previous scans, with new bilateral pleural effusions (Figures 2E–H). However, the progression of the metastases in other sites remained stable. Furthermore, the patient’s PS advanced from 3 to 4, with significant increases noticed in her CEA and CA19-9 levels (Figures 3A,B), thereby indicating a disease progression. Subsequently, palliative care, including anti-infective agents, pleural effusion management, oxygen therapy, and analgesics, was implemented. However, for patients with ERBB2-amplified mCRC who do not respond to multiple treatments, the available options for a subsequent treatment are primarily limited to Best Supportive Care (BSC). Despite the deteriorating condition, the patient and her family showed a strong desire for further treatment. After comprehensive discussions among our oncologists, the salvage anti-ERBB2 treatment plan was implemented on 3 November 2023, involving the use of Disitamab Vedotin (2.5 mg/kg IV infusion every 2 weeks) with oral Pyrotinib (320 mg daily). This therapeutic approach targets both the extracellular and intracellular domains of ERBB2, thereby enhancing anti-tumor efficacy. According to the CT results obtained on 28 November 2023, an efficacy assessment was performed which showed a partial response based on the Response Evaluation Criteria in Solid Tumors 1.1 criteria after two cycles of the specified targeted therapy (Eisenhauer et al., 2009) (Figures 2I–L). Concurrently, notable reductions in the levels of the two tumor markers CEA and CA199 were observed during the combination therapy (Figures 3A, B). Simultaneously, the patient’s symptoms of hemoptysis and extreme fatigue were consistently addressed following the switch to the most recent anti-ERBB2 therapy; this resulted in the improvement of the performance status score of 2. After completing five treatment cycles, no severe adverse effects such as hematotoxicity, hepatic dysfunction, pulmonary damage, or diarrhea, were observed. However, mild instances of nausea and fatigue were observed during the medication course. Presently, the patient remains stable and is undergoing further treatment in our hospital. A summary of the treatment regimens and clinical features is presented in Figure 4.

## Discussion

This case report presents a case of an ERBB2-amplified refractory mCRC patient’s partial response to a salvage treatment regimen of Disitamab Vedotin combined with pyrotinib, and tells how disease progression was continuously controlled in the patient. To our knowledge, this is the first case of use of Disitamab Vedotin with Pyrotinib regimen for ERBB2-amplified refractory mCRC, that showed encouraging efficacy.

The prognostic value of ERBB2 amplification in mCRC remains a debated topic. However, the findings from recent clinical trials indicate that some patients achieve partial response and survival benefits with anti-ERBB2 agents, including various combinations of monoclonal antibodies, antibody-drug conjugates (ADCs), and tyrosine kinase inhibitors (TKIs) (Strickler et al., 2022). The HERACLES-A study is a prospective, open-label, phase II trial that focused on targeting ERBB2 for therapeutic intervention. This salvage therapy employed a combination of trastuzumab and lapatinib that was given to 27 patients with KRAS wild-type and HER2-positive mCRC. The results indicated an objective response rate (ORR) of 28% and median progression-free survival (PFS) of 4.7 months, with no grade 4 or 5 adverse events (Sartore-Bianchi et al., 2016). The MOUNTAINEER trial, a global, open-label, phase 2 study, enrolled patients with HER2-positive, RAS wild-type unresectable or metastatic colorectal cancer to evaluate the effectiveness of combining tucatinib with trastuzumab, both of which target HER2. This treatment combination demonstrated an ORR of 38.1% despite causing adverse effects such as diarrhea, hypertension, and hepatic and renal impairment, all of which were considered acceptable and manageable for advanced-stage tumors (Strickler et al., 2023). Despite having limited sample size, both trials provided valuable evidence supporting the use of ERBB2 as a therapeutic target for the involved patients.

Findings of HERACLES-A and the patient’s medical history (Sartore-Bianchi et al., 2016) revealed that a salvage therapy involving trastuzumab and lapatinib was initially utilized. However, the patient developed significant pleural effusion, suggesting insufficient disease management. Given the current situation, it was exceptionally challenging to choose potential effective strategies for subsequent treatment lines. Based on the presence of ERBB2 amplification, the mechanism of the Disitamab Vedotin and pyrotinib, and the patient’s strong desire for life-extending rather than supportive care, a salvage treatment combining Disitamab Vedotin with pyrotinib was ultimately attempted, which unexpectedly demonstrated satisfactory effectiveness.

Although no prospective studies have been conducted to examine the efficacy of Disitamab Vedotin combined with Pyrotinib in mCRC patients harboring ERBB2 amplification, some studies on the use of these drugs in the treatment of HER2-positive tumors were undertaken. Recently, two single-arm studies (RC48-C005 and RC48-C009) have reported on the treatment of HER2-positive metastatic urothelial carcinoma patients with Disitamab Vedotin following the failure of systemic chemotherapy. The median PFS and overall survival (OS) in the studies were 5.9 months (95% CI, 4.3–7.2) and 14.2 months (95% CI, 9.7–18.8), respectively (Sheng et al., 2023). Additionally, a patient with HER2 2+ advanced gastric cancer and systemic metastasis, who had failed first-line treatment, achieved a 6-month PFS after receiving Disitamab Vedotin (Dai et al., 2022). Furthermore, a recent prospective observational study demonstrated promising anti-tumor effects in patients with HER2-positive and Ras wild-type mCRC following treatment with either Pyrotinib alone or in combination with trastuzumab (Zhou et al., 2024). In the subgroup that received pyrotinib and trastuzumab, patients achieved a median PFS of 8.6 months and an ORR of 50.0% while in the pyrotinib monotherapy group, the median PFS and the ORR were of 5.5 months and 25.0%, respectively (Zhou et al., 2024). Diarrhea, peripheral sensory neuropathy, increased AST (Aspartate Aminotransferase) levels, leukopenia, hand-foot syndrome, and fatigue were commonly observed adverse reactions in trials or cases involving treatment with the two drugs (Deeks, 2021; Blair, 2018). However, in the present case, the patient only experienced mild nausea and fatigue, which could possibly be attributed to the extensive metastases characteristic of advanced colorectal cancer.

The primary limitation of this case report is its singular nature. Though we speculate that the observed disease control may be attributed to the administration of Disitamab Vedotin and Pyrotinib, further basic and clinical trials are needed to confirm the efficacy of this combination in mCRC patients with ERBB2 amplification.

Overall, we report a case of a female patient with refractory metastatic colorectal cancer harboring ERBB2 amplification. This patient demonstrated a partial response to salvage therapy combining Disitamab Vedotin with Pyrotinib, resulting in significant improvements in continuous disease control and tumor marker levels. Because of the high ORR of ERBB2-targeted therapy in salvage treatment for colorectal cancer, it is suggested to further explore and improve ERBB2-targeted therapy in future studies.

## Data Availability

The original contributions presented in the study are included in the article/Supplementary Material, further inquiries can be directed to the corresponding author.
